# Electroacupuncture-Induced Neuroprotection against Cerebral Ischemia in Rats: Role of the Dopamine D2 Receptor

**DOI:** 10.1155/2013/137631

**Published:** 2013-11-21

**Authors:** Ming-Shu Xu, Shu-Jing Zhang, Dan Zhao, Cheng-Yong Liu, Chang-Zhi Li, Chun-Yan Chen, Li-Hui Li, Ming-Zhe Li, Jia Xu, Lin-Bao Ge

**Affiliations:** ^1^Neurobiology Laboratory of Brain, Shanghai Research Institute of Acupuncture and Meridians, Shanghai 200030, China; ^2^Shanghai Research Institute of Qigong, Shanghai 200030, China; ^3^Jiangsu Provincial Hospital of Traditional Chinese Medicine, Nanjing 210029, China; ^4^Yueyang Hospital of Integrative Chinese & Western Medicine Affiliated to Shanghai University of TCM, Shanghai 200437, China; ^5^Shanghai Research Center of Acupuncture and Meridians, Shanghai 201203, China

## Abstract

*Background*. Cerebral ischemia is known to produce brain damage and related behavioural deficits, including memory deficits and motor disorders. Evidence shows that EA significantly promotes recovery of neurological function and thus improves quality of life. *Objective*. Evidence exists for the involvement of catecholamines in human neuroplasticity. A better understanding of dopaminergic (DAergic) modulation in this process will be important. *Methods*. A total of 72 adult male Sprague-Dawley (SD) rats were divided into 6 groups: normal, model, EA, spiperone group, EA + spiperone group, and pergolide. The middle cerebral artery occlusion (MCAO) model was used in all 6 groups except the normal group. A behavioural assessment was conducted at 1, 3, 5, and 7 days after MCAO. The percent of brain infarct area was also determined 7 days after MCAO. Tyrosine hydroxylase (TH) and growth-associated protein 43 (GAP-43) fluorescence double labeling was performed in the striatum. *Results*. In this study, we found that EA at Fengchi (GB20) acupoints resulted in marked improvements based on a behavioural assessment. Both TTC staining and GAP-43 immunofluorescence labeling results showed that EA treatment reduced ischemia injury and promoted neuroplasticity compared with the model group. The D2R-selective agonist, pergolide, showed similar results, but these results were reversed by the D2R-selective antagonist, spiperone. We also found that there were more colocalization and expression of GAP-43 and TH in the EA and pergolide groups than those in the other groups. *Conclusion*. These results suggest that the neuroplasticity induced by EA was mediated by D2 autoreceptors in DAergic neurons.

## 1. Introduction

Cerebrovascular injury is one of the most prevalent diseases in the world, especially in developed countries and developing countries with continuously increasing standards of living, such as China. Stroke can be either ischemic or hemorrhagic, but more than 80% of stroke cases are caused by cerebral ischemia [1].

Cerebral ischemia is known to produce brain damage and related behavioural deficits, including memory deficits and motor disorders. Middle cerebral artery occlusion reportedly occurred in 10–15% of stroke patients [2]. The main areas affected by middle artery occlusion are the cerebral cortex, the hippocampus, and the striatum [3]. Memory and motor deficits are associated with interruption of blood flow to these areas [4–8].

Survivors of stroke are often affected by serious, long-term disabilities, including paralysis and disruption of higher cognitive functions, such as speech and memory. Some patients may even have mental disorders, such as depressive symptoms. Individuals with such disabilities often require extensive long-term care by health care professionals and family. 

Due to the high and wide-ranging social impact of cerebrovascular disease, there is great interest in researching methods to increase the cure rate of cerebrovascular diseases, reduce the financial burden of both government agencies and affected families, and improve the quality of life of ischemic patients. 

Although many neuroprotective agents have been proven to reduce infarction volume and improve neurological recovery in basic research with animal stroke models, few have shown positive effects in clinical trials [9, 10]. There is a wide gap between current treatments and our expectations. Currently, no clinical modality has demonstrated promising efficacy in terms of stroke treatment. Therefore, new strategies should be developed to establish better preventative measures and treatments for this serious disease.

Traditional Chinese medicines (TCMs) have been used successfully for centuries to treat a wide variety of ailments and have attracted increasing attention from industry and academia in China [11–13].

EA is a therapy based on traditional acupuncture, combined with modern electrotherapy. Acupuncture to different brain areas is known to have beneficial effects, and EA has been seen as an improvement on traditional acupuncture. Evidence shows that EA significantly promotes recovery of neurological function and thus improves quality of life [14].

Numerous studies have confirmed that acupuncture can be beneficial to patients during convalescence from ischemic apoplexy [15]. Animal experiments [16] have also demonstrated that acupuncture can accelerate the restoration of function and help heal the cerebral tissue lesion during cerebral ischemia-reperfusion. Dopamine plays an important role in this process.

Some key concepts for developing effective rehabilitation interventions are the heterogeneity of mechanisms underlying stroke as well as the plastic processes leading to recovery of function after neuronal injury.

DAergic neurons are subject to modulation by a variety of factors. Some of the factors involved in intrinsic regulation of central DA neurotransmission during physical activity include TH, D1, and D2 receptors.

Evidence exists for the participation of catecholamines in human neuroplasticity. Regional discrepancies observed in the action of dopamine on synaptic plasticity [17] could be explained by differences in dopamine content and dopamine receptor subtype distribution, resulting in differences in the level of dopamine receptor activation during LTP or LTD induction.

Dopamine neurons are important for neuroplasticity after ischemia. Electroacupuncture (EA) significantly promotes recovery of neurological function. Previous studies have shown that dopamine D2 receptor plays a importance role in the induction of neuroplasticity. The recovery of neurological function is interrelated and inextricably linked with neuroplasticity. Given the recovery of EA and the importance of the dopamine system in neuroplasticity after ischemia, we hypothesized that Dopamine D2 receptor may play an important role in the neuroprotection induced by EA. To reflect the progression of the disease and recovery, the behavioural observations should be combined with histopathology. A better understanding of DAergic modulation will be important for understanding neuroplastic processes after ischemia.

## 2. Materials and Methods

### 2.1. Animals

A total of 72 adult male Sprague-Dawley (SD) rats (8-9 wks of age, 300 ± 20 g, Shanghai Laboratory Animal Center, Chinese Academy of Sciences, Shanghai, China) were raised in groups of 4–6 per cage under controlled conditions (23 ± 1°C, 50% ± 10% relative humidity, 12/12 hr alternate light/dark cycles, and food and water ad libitum) for at least 1 week before the experiments. All animals were handled with care to prevent infection and minimize stress. 

The experimental protocols were approved by the Institutional Animal Care and Use Committee. The minimum number of animals and duration of observations required to obtain reliable data were used.

### 2.2. Environmental Adaptation and Grouping

**Figure 1:**
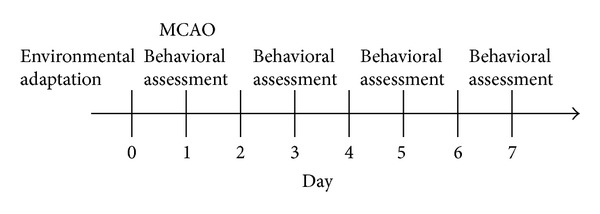
Experimental procedure. Normal group: behavioural assessment, but no MCAO. The other group: behavioural assessment and MCAO. EA, pergolide, or spiperone treatment was given each day after MCAO.

Based on a random number table, the rats were divided into 6 groups: normal, model, EA, spiperone group, EA + spiperone group, and pergolide group, with 12 rats in each group. The middle cerebral artery occlusion (MCAO) model was established in all 6 groups except the normal group. The D2R antagonist, spiperone, was delivered by peritoneal injection in the D2R antagonist group and the EA + spiperone group once a day for 7 days. The D2R agonist, pergolide, was delivered by peritoneal injection in the pergolide group once a day for 7 days. EA was applied once a day for 7 days in the EA group and the EA + spiperone group. In this study, the doses of spiperone and pergolide were selected based on our pilot study and earlier reports. Behavioural assessments were conducted 1, 3, 5, and 7 days after MCAO. The brain infarct area was also determined 7 days after MCAO. The schedule for drug treatment, surgery, and behavioural testing is shown in Figure 1.

### 2.3. Reagents

10% chloral hydrate; gentamycin sulfate injection; 1% heparin sodium solution; D2R agonist pergolide (P8828, Sigma, America); D2R antagonist spiperone (108587, Sigma, America); GAP-43 antibody (G9264, Sigma, America); TH antibody (ab6211, Abcam, America).

### 2.4. Instruments

SD-78 bipolar coagulator; G6805-2 electroacupuncture instrument (Shanghai Huayi Medical Instrument Factory); YP1201N electronic balance (Shanghai Precision Scientific and Balance Instrument Factory); 40-90-8C rat temperature control pad (Frederick Haer, America); acupuncture needles (0.25 mm in diameter and 13 mm in length, Suzhou Acupuncture Supplies Factory).

### 2.5. Induction of the MCAO Rat Model

Plug lines were prepared with 3.0 cm nylon monofilament (4-0) suture (DG, America). The tips were rounded by heating near a flame and then washed with normal saline and placed in tubes filled with heparin sodium 1% solution. 

The rat model of middle cerebral artery ischemia-reperfusion was established according to the literature [1]. Briefly, rats were anesthetized with chloral hydrate (400 mg/kg, i.p.). The right common carotid artery (CCA) and internal carotid artery (ICA) were exposed via a midline incision in the neck. The pterygopalatine artery was ligated close to its origin. The nylon filament suture was advanced from the right external carotid artery, through the CCA and up to the ICA for a distance of 18  ±  0.5 mm to block the origin of the middle cerebral artery (MCA), until a mild resistance was felt. The right MCA was occluded for 90 min. After that, cerebral blood flow (CBF) was restored by withdrawal of the nylon thread. The rectal temperature was maintained at 37°C–37.5°C during and after surgery. The sham group underwent the same surgical procedure without insertion of the nylon thread [18, 19].

### 2.6. Behavioural Assessment

Researchers and an assistant who was not involved in this experiment scored the neurological deficits.

#### 2.6.1. Index of Neurological Deficits Test

Neural function defect score (NFDS) standard [3]: rats show no asymmetric activity, 0 points; rats are unable to stretch the left forelimb when the tail is lifted, 1 point; the left forelimb could not straightly downwards accompanied by the abduction of the left shoulder, 2 points; left forelimb is close to the chest wall, 3 points; rats turn left in free activities, 4 points; accompanied by obvious left-front paw pushing back, 5 points; rats can only rotate around the origin to the left, 6 points; limbs cannot support the body weight on the left side, and the rats can only lie on the left side, 7 points.

#### 2.6.2. Balance Beam Test (BBT)

Rats were placed on a wooden bar of 300 mm × 25 mm. Rats can maintain balance with four feet and walk across the wooden bar, 0 point; rats can't walk across the wooden bar, but can maintain balance with four feet, 1 point; rat's claws grip the side of the wooden bar or rat's body shakes on the bar, 2 points; one limb slips from the bar, 3 points; two limbs slip from the bar, 4 points; rats try to keep their balance but slip, 5 points; rats fail to keep their balance, hang on the bar and fall down, 6 points; rats fall down directly without trying to keep their balance, 7 points [20].

#### 2.6.3. Limb Placement Test

In this study, sensorimotor integration was evaluated over a 7-day period by an investigator blind to the rats' treatment regimen. In the forelimb placement test (FPT), animals were held gently by the torso and moved slowly toward a table top until the dorsal forepaw surface barely touched the edge. Normal animals rapidly place their forelimb on the table top. Performance was scored between 0 (normal) and 10 (maximal impairment). Similarly, the hindlimb placing test (HPT) evaluated the animal's ability to place the hindpaw on a table in response to light stimulation and was scored on a 0–6 scale [20, 21].

### 2.7. EA Intervention Scheme

The location of the rat Fengchi (GB 20) is similar to that in the human body under the occipital bone in the hollow between the trapezius and sternocleidomastoid muscles. Two stainless steel needles were perpendicularly inserted 8 mm into the Fengchi (GB 20) and connected to the EA instrument. The parameters were as follows: frequency of 2 Hz, continuous wave, and current intensity of 3.0 mA (oscilloscope detection), with mild jittering of the rat auricle. The EA lasted for 20 min, was stopped for 10 min, and then resumed for another 20 min.

### 2.8. Infarct Area Assessment

Following neurological function evaluation, 6 rats in each group were deeply anesthetized by an intraperitoneal dose of 400 mg/kg chloral hydrate and then decapitated. Each brain was removed and sliced in 2 mm sections using a rodent brain matrix slicer (RBM-4000C; ASI Instruments, Warren, MI, USA). Sections were stained with 2,3,5-triphenyltetrazolium chloride (TTC) (Nanjing Green Synthesis Biochemical Co., Ltd., Nanjing, Jiangsu, China). The percent of infarct area of the entire brain represented the degree of cerebral infarction. Serial coronal sections were prepared and soaked in 2% TTC phosphate buffer at 37°C for 10 minutes in the dark. Normal brain tissues were stained red, while infarct tissues were not stained (white). The sections were soaked in 4% paraformaldehyde phosphate buffer for 30 minutes, arranged in order, and scanned. Areas of red and white staining were measured using a computer colour multimedia image analysis system (Image J 1.46R, NIH, USA). The percent of infarction is given by the equation: %infarct area = infarct area/total area of slice × 100 [22].

### 2.9. Double Immunofluorescent Labeling

Following anaesthesia with chloral hydrate (60 mg/kg body weight), 6 rats in each group were transcardially perfused with fixative containing 4% paraformaldehyde in 0.1 M phosphate buffer (pH 7.3). The brains were removed and stored overnight in the same fixative. They were infiltrated with 30% sucrose solution and kept at 4°C. The specimens were rapidly frozen and sectioned with a vibratome (30 *μ*m sections) on a cryostat. In order to examine the relation between TH and GAP-43 in the striatum, fluorescence double labeling was performed. After washing in PBS (pH 7.3), striatal sections were incubated for 30 min at room temperature in 10% goat serum diluted in PBS. They were then incubated for 48 h at 4°C with a mixture of antisera against TH and GAP-43 diluted 1 : 1000 and 1 : 100 in PBS, respectively. After washing in PBS, they were incubated for 1 h at room temperature with a mixture of pig antisera against IgG of mouse antisera conjugated to Cy3 diluted 1 : 100 and goat antisera against IgG of rabbit antisera conjugated to fluorescein isothiocyanate (FITC) diluted 1 : 100 in PBS. DAPI was used as an additional nuclear counterstain. The sections were mounted on glass slides. Image analysis of the double immunofluorescent labeling was performed by a SP5-AOBS confocal laser-scanning microscope (Leica Microsystems, Mannheim, Germany) through a 20 × 0.5 NA air objective and a 40 × 1.2 NA oil-immersion objective, using laser excitation at 488 and 561 nm. Images were assembled into montages with Image J software (NIH; http://rsb.info.nih.gov/ij/) and Adobe Photoshop 7.0 (Adobe Systems, Mountain View, CA, USA). The size distribution of positive cell profiles was determined using NIH Image software. The somas of neurons of interest were outlined manually, and their sizes were determined. Only neurons with a distinguishable nucleus in the section were counted [23, 24].

### 2.10. Statistical Analysis

Data are expressed as mean ± SD. Data from all groups were compared using a one-way ANOVA followed by post hoc analysis for significance with the Student-Newman-Keuls multiple comparison test. A probability value of less than 0.05 was considered statistically significant.

## 3. Results

### 3.1. Behavioural Assessment

**Figure 2:**
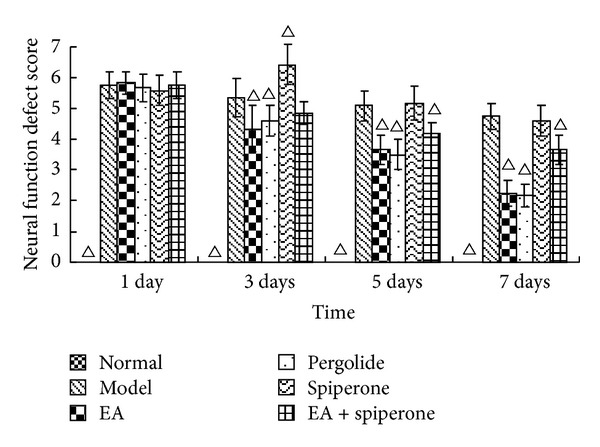
NFDS changes in all groups except the normal group 1, 3, 5, and 7 days after MCAO. The columns represent the normal, model, EA, pergolide, spiperone, and EA + spiperone groups, respectively. The score for the normal group on each day was 0 (data not shown), Δ*P* < 0.05 versus model group at the same time point.

**Figure 3:**
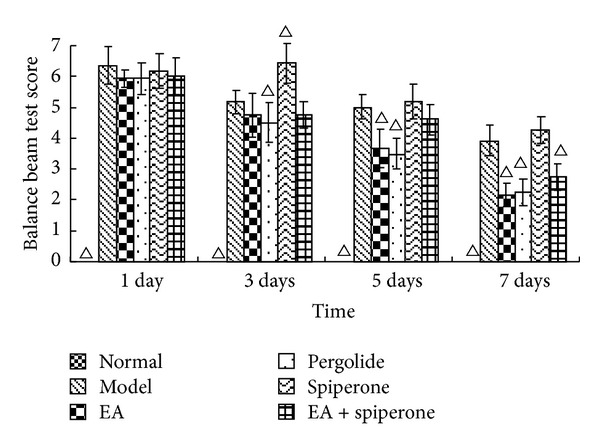
BBT score changes for all groups except the normal group 1, 3, 5, and 7 days after MCAO. The score for the normal group on each day was 0 (data not shown). Δ*P* < 0.05 versus model group at the same time-point.

All 72 rats were included in the results analysis, without any animals being lost in the course of the experiment. NFDS and BBT scores may reflect the neurological deficit and the impaired balancing ability in rat brain function. Before MCAO, the NFDS and BBT scores for all groups were 0. In the experiment, we observed that the neurological deficit was aggravated after MCAO. The situation was stable until 24 h after reperfusion. NFDS and BBT scores taken 1, 3, 5, and 7 days after reperfusion are shown in Figures 2 and 3. There were significant differences in NFDS and balance beam test scores before and after MCAO (*P* < 0.05). After the EA and pergolide interventions, the NFDS and BBT scores in the EA group and the pergolide group had improved more significantly than those in the model group (*P* < 0.05). The NFDS and BBT scores in the spiperone group had worsened 3 days after reperfusion and were significantly different from the model group. The NFDS and BBT scores in the EA + spiperone group had noticeably improved compared with those in the spiperone only group. (Figures 2 and 3).

**Figure 4:**
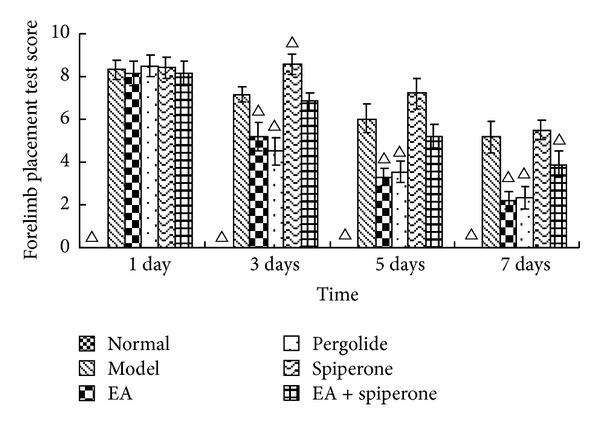
FPT score changes in all groups except the normal group 1, 3, 5, and 7 days after MCAO. The score for the normal group on each day was 0 (data not shown). Δ*P* < 0.05 versus model group at the same time-point.

**Figure 5:**
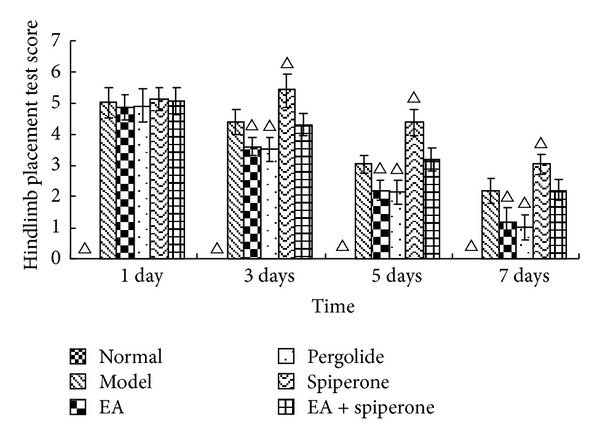
HPT score changes in all groups except the normal group 1, 3, 5, and 7 days after MCAO. The score for the normal group on each day was 0 (data not shown). Δ*P* < 0.05 versus model group at the same time-point.

FPT and HPT score changes appear similar to the changes in NFDS and BBT at 1, 3, 5, and 7 days after modeling. The FPT and HPT scores were both 0 in the normal group on each day. The neurological deficit was aggravated after MCAO. EA and pergolide improved the score compared with the model group (*P* < 0.05). The spiperone group showed decreased FPT and HPT scores 3 days after reperfusion; however, these scores noticeably improved in the EA + spiperone group. (Figures 4 and 5).

### 3.2. TTC Staining

**Figure 6:**
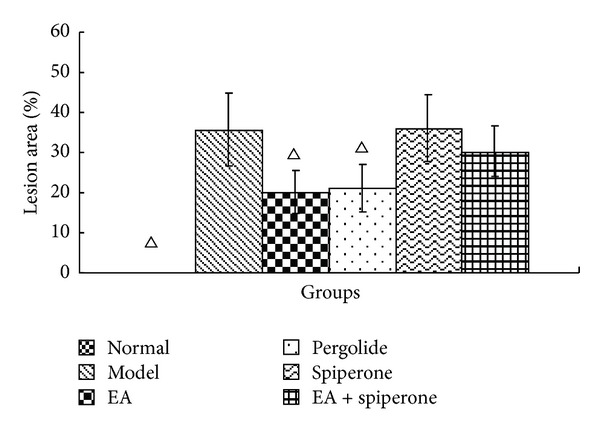
Percent lesion area in the contralateral hemisphere ($\overset{-}{X}\pm \text{SD}$) (*n* = 6). The percent lesion area in the normal group was 0 (data not shown). Δ*P* < 0.05 versus model group at the same time-point.

**Figure 7:**
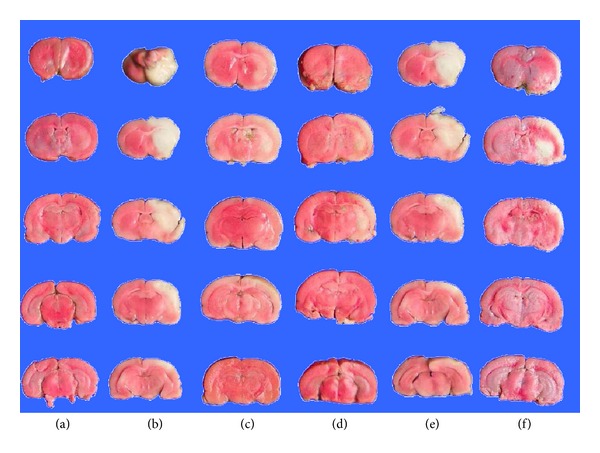
Effect of EA, pergolide, and spiperone on brain infarct area. Brain infarct area was determined using TTC staining. (a) Normal, (b) model, (c) EA, (d) pergolide, (e) spiperone, (f) EA + spiperone. Sections are arranged from rostral (top) to caudal (bottom).

TTC staining may reflect the neurological deficit in the rat brain. There were significant differences in lesion area between rats in the normal and model groups (*P* < 0.05). After interventions with EA and pergolide, the lesion area in the EA and the pergolide groups was reduced noticeably compared with the model group (*P* < 0.05). The area of the lesion in the spiperone group had worsened and showed no significant difference from the model group. In the spiperone and EA group, the area of the lesion was reduced, but no significant difference was found when compared with the spiperone group (Table 1 and Figures 6 and 7).

### 3.3. Double Immunofluorescent Labeling (GAP-43 and TH Immunocolocalization)

**Figure 8:**
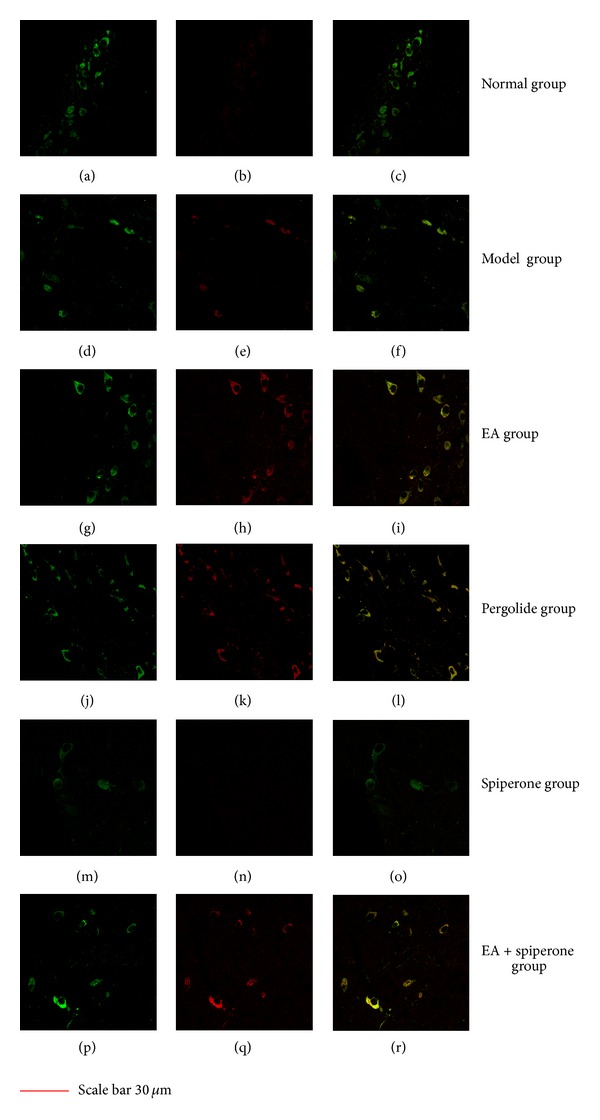
Fluorescence double staining of TH (tyrosine hydroxylase) and GAP-43 (growth-associated protein 43).

As shown in Figure 8, colocalization experiments indicated that TH-positive cells (green) and GAP-43-positive cells (red) were colocalized in some neurons of the striatum. The double immunostaining revealed that only a third of TH-positive cells produced GAP-43, meaning that the colocalization was only partial. EA and pergolide led to increased GAP-43 expression in DAergic neurons 7 days after the onset of ischemia compared with the model group (*P* < 0.05) (data not shown) (Figure 8, resp.). There was no variation between the two groups of animals (data not shown). The colocalization of both GAP-43 and TH decreased in the spiperone group compared to the EA and pergolide groups (*P* < 0.05). Indeed, no GAP-43/TH double-labeled cells were observable in the spiperone group. Such an effect could be partly reversed by EA. No significant differences in co-localization were found between the model group and the EA + spiperone group (*P* > 0.05).

Photomicrographs show neurons in the rat striatum after double labeling with TH antiserum (shown in green) and GAP-43 antiserum (shown in red) of normal Figures 8(a)–8(c), model Figures 8(d)–8(f), EA Figures 8(g)–8(i), pergolide Figures 8(j)–8(l), spiperone Figures 8(m)–8(o), and EA + spiperone groups Figures 8(p)–8(r). The immunoreactivity of TH and GAP-43 decreased after spiperone treatment while EA or pergolide treatment increased the immunoreactivity of these neurons. There was colocalization of TH and GAP-43 (yellow) in some neurons in the model Figure 8(f), EA Figure 8(i), pergolide Figure 8(l), and EA + spiperone Figure 8(r) groups. Scale bar is 30 *μ*m.

## 4. Discussion

Although two systematic reviews have indicated that there is no enough evidence to support the claim that acupuncture has a positive effect on functional recovery after stroke [15, 25], certain clinical studies have revealed that acupuncture may be an effective therapy for ischemic stroke [26]. Many recent clinical trials have verified that acupuncture can improve balance function [27] and spastic states [28] in stroke patients reduce muscle spasticity, and improve motor function in chronic stroke survivors with moderate or severe muscle spasticity [29]. Lewith et al. [30] systematically researched and reviewed the literature, looking at how acupuncture affects brain activation as measured by functional magnetic resonance imaging and positron emission tomography, and found that specific and largely predictable areas of brain activation and deactivation occur when traditional Chinese acupuncture is applied to certain specific acupuncture points. In addition, 46% of stroke survivors in the United States use some form of complementary and alternative medicine (CAM) therapy. Acupuncture was the most frequently used CAM therapy in stroke survivors [31]. However, the beneficial effects of acupuncture in stroke patients required more high-quality evidence [32]. Integrated with electrotherapy, EA is conducted by inserting acupuncture needles into acupoints and then changing electrical stimulation parameters, including the stimulation frequency, current intensity, pulse width, and pulse interval. Thus, EA not only inherits the benefits of traditional acupuncture but also combines the physiological effects of electric stimulation [33]. 

A large number of animal studies have shown that EA can reduce neural apoptosis, promote cell proliferation, increase cerebral blood flow (CBF), and improve neurological function after stroke [34–36]. These results provide some evidence for further translational studies.

Recent evidence suggests that nitric oxide, serotonin, catecholamines, and amino acids such as glutamate and *γ*-aminobutyric acid (GABA) are mediators of the neurobiological effects of acupuncture, but at present their role is still poorly understood. Therefore, acquiring further information about the neurobiological mechanisms of acupuncture should be the aim of research in the future [19].

There have often been discrepancies between neuroprotective drug studies in animals and clinical studies in humans. Many drugs appear to work in animal experiments but fail in clinical studies. In preclinical studies, determination of neuroprotection has relied heavily on assessment of infarct volume (instead of functional outcomes), short-term (instead of long-term) end points, short (instead of extended) time windows for drug administration, and protection of cerebral gray matter (instead of both gray and white matter). Current methodologies have been reevaluated. New concepts in ischemic pathophysiology should encourage researchers to think beyond the hyperacute phase of ischemia and consider multiagent therapies that exploit the brain's capacity for neuroplasticity and repair [37]. The reorganization of functional areas after ischemia and methods for assessing the restoration of function should compensate for the above shortcomings.

Functional restoration after a stroke relies on neuroplasticity, and neuroscience research has increasingly focused on studying this neuroplasticity [38]. Neuroplasticity has been defined as the ability of neurons and circuits to modify (1) their functional activity (short- or long-term potentiation/depression) and/or (2) their synaptic organization in accordance with variations in activity [39, 40]. Neuroplasticity is present at all points in an individual's lifespan: development, adulthood, after injury, during memory formation and/or learning, and so forth. 

Even though neuroplasticity is especially intense and a key process during development, it is still present and necessary in adulthood [41]. The maintenance of neuroplastic activities is also necessary for nerve recovery after damage, such as stroke [42].

Behavioural examinations are the main means to determine early neuronal death after cerebral ischemic injury and are also a way to evaluate the restoration of neurological function caused by neuroplasticity. Since Longa et al. [43] developed their criteria for the evaluation of neurological deficits caused by cerebral ischemia in animals, this neurological deficit score has improved greatly and is now broadly applied to estimate the curative effects of diverse treatments on many kinds of animals [44]. Our research showed a rapid decline in rat behavioural scores after MCAO. BBT and NFDS scores were significantly improved after interventions with EA and D2R agonists, which indicate that these compounds promote recovery of neurological function after cerebral ischemic injury. FPT and HPT score changes appeared similar to the NFDS and BBT score changes seen 1, 3, 5, and 7 days after modeling. Spiperone decreased the FPT and HPT scores at 3 days after reperfusion. FPT and HPT scores were improved with spiperone and EA. EA has been shown to promote the expression of neurotrophic factors [15], effectively improve functional neural impairment, and promote brain plasticity. Studies have shown that D1R can enhance excitotoxicity and increase nerve damage [4].

It is necessary to combine behavioural observations with histopathology in order to reflect the progression of the disease and treatment more objectively. The results of TTC staining suggested that EA and D2R agonists could decrease the infarct area. Furthermore, D2R antagonists could increase the infarct area. The infarct area of each group with MCAO should be similar, but in fact, there was a significant difference between the EA group and the model group, similar to the difference seen between the pergolide group and the model group. These results suggest that EA and pergolide might save the ischemic penumbra and decrease the infarct volume through reducing or stopping the secondary injury after cerebral ischemia and reperfusion. On the contrary, spiperone aggravated the secondary injury. EA was able to partially reduce the injurious effects of spiperone. Our results indicate that D2R plays an important role in the injury-reducing effect of EA treatment for ischemia and reperfusion. 

In addition to reducing injury after ischemia, DAergic neurons are important for neuroplasticity after ischemia. Research has shown that endogenous DA plays a physiological role through DR and TH. Dopamine receptors are classified into two categories: D1-like and D2-like. D1 receptors function primarily as postsynaptic heteroreceptors on non-DAergic neurons. In contrast, D2 receptors have dual roles in DA neurotransmission as autoreceptors and postsynaptic receptors [45].

A better understanding of DAergic neuroplasticity modulation will be important for understanding the rehabilitation processes, not only in animals but also in humans. Evidence exists for the participation of catecholamines in human plasticity. As previously shown, amphetamine (a catecholamine reuptake blocker) stabilizes use-dependent motor cortex plasticity, accelerates recovery of motor function in stroke patients, and improves learning and consolidation of verbal material [46–48]. Recent work has demonstrated that application of a single dose of levodopa significantly improves the formation of a motor memory in healthy subjects as well as in chronic stroke patients [49].

There is agreement that dopamine increases NMDA currents through D1 receptors [50]. Both D1 and NMDA receptors were suggested to contribute to the mechanisms of LTP by inducing the accumulation of cAMP and the activation of PKA [51].

The importance of D2 receptors for the induction of neuroplasticity has been described in animal experiments [52]. Moreover, it was demonstrated that D2 receptors determine the direction of neuroplastic changes in the striatum of mice [53]. In healthy humans, the D2 antagonist, SULP, and the predominant D2 antagonist, haloperidol, [54] impaired learning. This adds further behavioural evidence to the importance of D2 receptors for neuroplasticity in humans.

To further evaluate the role of DAergic neurons in neuroplasticity, cellular localization of GAP-43 formation was assessed in DAergic neurons by double immunostaining experiments (immunohistofluorescence for TH and GAP-43) performed after 7 days of ischemia. According to previous research, tyrosine hydroxylase was widely accepted as the marker of DAergic neurons [55]. GAP-43 is a calmodulin-binding phosphoprotein found in growing axons and growth cones of developing neurons and also in regenerating axons. The expression of GAP-43 might indicate the existence of a regeneration or neuroplasticity process, such as long-term potentiation. GAP-43 is considered a useful marker of developing neural connections and neuroplasticity or regenerating nerve fibres [56].

EA and pergolide led to an increase in GAP-43 expression in DAergic neurons. Spiperone decreased the co-localization of both. This effect of spiperone could be partly reversed by EA. The results of this study are additional evidence for a plasticity-enhancing effect of D2 receptor activity after ischemia and favour the hypothesis that for rehabilitation after stroke, DAergic treatment could enhance plastic reorganization of cortical areas. Indeed, some studies report a beneficial effect of DAergic medication, when combined with motor learning paradigms or physiotherapeutic rehabilitation after stroke [57].

Previous research has shown that transcranial direct current stimulation (tDCS) leads to modulation of cortical network plasticity by application of weak direct currents through the surface of the scalp. DA is necessary to induce this kind of neuroplasticity and it also strengthens and consolidates it [51, 57]. The EA treatment used in our research applied weak direct currents to acupoints near the scalp and produced a similar result. Moreover, our results indicate that the neuroplasticity induced by EA is mediated by a D2 autoreceptor in DAergic neurons.

In conclusion, the present findings imply that DAergic neurons play an important role in the induction of neuroplastic changes after ischemia, through a D2-like receptor, and that this receptor plays an important role in EA treatment for ischemia. Although important progress has been made in the comprehension of DAergic neuron function, the identification of the neuroplastic mechanisms of DAergic neurons in the CNS is a prerequisite in determining future research and rehabilitation strategies. A targeted protection of DAergic neurons could represent a novel and exciting approach to potentiate poststroke neuroregenerative responses [37].

## Figures and Tables

**Table 1:** Percent lesion area in the contralateral hemisphere ($\overset{-}{X}\pm \text{SD}$) (*n* = 6).

Group	Lesion area, %
Normal	0
Model	35.7 ± 9.1
EA	20.1 ± 5.2
Pergolide	21.1 ± 6.1
Spiperone	35.9 ± 8.4
EA + spiperone	30.2 ± 6.3
